# Characteristics and predictors of breast milk iodine in exclusively breastfed infants: Results from a repeated-measures study of iodine metabolism

**DOI:** 10.3389/fnut.2022.1017744

**Published:** 2022-11-09

**Authors:** Wenxing Guo, Wen Wu, Min Gao, Ying Yang, Elizabeth N. Pearce, Shaohan Li, Zhiyuan Ren, Naifan Zhang, Kexin Zhang, Ziyun Pan, Wanqi Zhang

**Affiliations:** ^1^Department of Nutrition and Food Hygiene, School of Public Health, Tianjin Medical University, Tianjin, China; ^2^Section of Endocrinology, Diabetes, and Nutrition, Boston University School of Medicine, Boston, MA, United States; ^3^Department of Endocrinology and Metabolism, Tianjin Medical University General Hospital, Tianjin, China; ^4^The Key Laboratory of Hormone and Development (Ministry of Health), Tianjin Institute of Endocrinology, Tianjin Medical University, Tianjin, China

**Keywords:** breast milk iodine, breastfed infants, urine iodine excretion, iodine metabolism study, iodine intake

## Abstract

**Background:**

The iodine supply of exclusively breastfed infants entirely depends upon breast milk. Changes in breast milk iodine affect infants’ iodine nutritional status. This study aimed to comprehensively assess the characteristics and predictors of breast milk iodine concentration (BMIC).

**Materials and methods:**

This 7-day iodine metabolism experiment was conducted in 25 exclusively breastfed mother-infant pairs. The duplicate portion method was used to measure the mother’s daily iodine intake from foods and water, and maternal 24-h urine excretion was assessed. We recorded the number of breastfeeds per mother per day and collected breast milk samples before and after each feeding.

**Results:**

The median [quartile (Q)1–Q3 range] of BMIC was 115 (86.7, 172) μg/L. The BMIC before breastfeeding was generally higher than that after breastfeeding. Time-sequential analysis found that morning BMIC was most highly correlated with the prior day’s iodine intake. Breast milk samples taken in the afternoon or after midnight are closer to the median level of BMIC throughout the day. The number of breast milk samples needed to estimate the iodine level with 95% CI within precision ranges of ± 20% was 83 for a population, 9 for an individual, and 2 for an individual’s single day. Maternal total iodine intake (TII) and urine iodine were significantly associated with BMIC. 24-h urinary iodine excretion (24-h UIE) was found to be the best predictive indicator for the BMIC (β = 0.71, 95% CI: 0.64, 0.79).

**Conclusion:**

BMIC is a constantly changing indicator and trended downward during each breastfeeding. Breast milk samples taken in the afternoon or after midnight are most representative. BMIC was significantly associated with recent iodine intake. Maternal 24-h UIE was the best predictor of BMIC.

## Introduction

Iodine, an essential micronutrient, is required by humans for the synthesis of thyroid hormones. Although suboptimal iodine intake can lead to a broad spectrum of disorders throughout life (1), adequate iodine intake is most critical in the early stages of development, as the infant’s normal neurodevelopment and growth are extremely dependent on the iodine supply. In the first 1,000 days of life, iodine deficiency can cause hypothyroidism and irreversibly impair neurodevelopment (2–4). The World Health Organization (WHO) recommends exclusive breastfeeding during the first 6 months of life, and breast milk is the only source of iodine for exclusively breastfed infants (5). Breast milk iodine thus reflects the iodine nutrition of both mothers and their breastfed infants (5–7).

Several factors such as maternal iodine status and duration of breastfeeding may influence breast milk iodine concentration (BMIC) (5). However, existing studies have only collected spot urinary iodine and breast milk iodine for comparisons, without comprehensively studying the relationship between breast milk iodine and maternal iodine intake (6, 8). Two recent reviews pointed out that the majority of studies on breast milk iodine have collected colostrum or mature milk only once and have had small sample sizes (5, 9). No study protocol involved whole-day breast milk sample collection. Furthermore, although some studies have shown large variations in BMIC (7), there are limited data on intra-individual, inter-individual, and daily variations in BMIC. Currently, studies that comprehensively summarize the characteristics of breast milk iodine are lacking.

The objectives of the study were to comprehensively assess the characteristics of BMIC and to identify parameters associated with breast milk iodine.

## Subjects and materials and methods

### Study setting and sample

**FIGURE 1:**
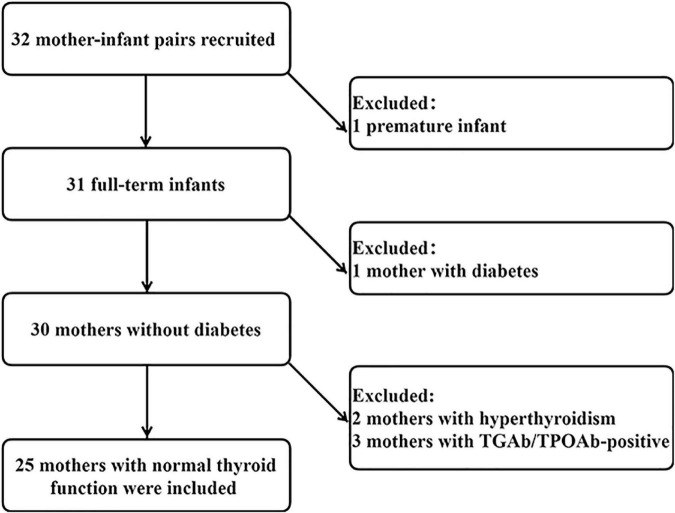
Flowchart of the study.

This study was performed in Gaoqing Country of Zibo City in Shandong Province, China. The study was home-based. Inclusion criteria were as follows: (1) lactating women aged 20 to 45 years; (2) no dietary restrictions; (3) no history of thyroid disease; (4) infants of singleton pregnancy; (5) delivery at full term and normal fetal birth weight (2.5–4 kg); (6) infants 0 to 6 months of age; and (7) exclusive breastfeeding. All infants and mothers were in good health. Initially, 32 volunteers were recruited, and after preliminary screening, 25 health mother-child pairs were included in the final analysis. A flowchart is presented in Figure 1. The Medical Ethics Committee of Tianjin Medical University approved the research protocols (ethics approval number TMUhMEC2020033). Subjects provided written informed consent before the study. This study was registered at www.clinicaltrials.gov as NCT04492657.

### Study design

Iodine intake (diet and water) and 24-h urinary iodine excretion (24-h UIE) of the lactating women were assessed. For infants, we recorded number of feedings per day and retained breast milk samples from each feeding. Researchers who underwent professional training measured infant length and weight on the day of the study. Weight measurements were performed using a special infant weight scale. The instrument was placed on a horizontal table and zeroed before measurement, and the infant was placed on the scale after removing the infant’s outer clothing, shoes and hats, and their weight was weighed and recorded. Then the infant was placed supine on a horizontal bed, kept by the investigator on the right side of the infant, holding the infant’s knees with the left hand and keeping the legs straight. The other person used a steel ruler to measure the length of the child from the heel to the top of the head. A detailed questionnaire was used to collect information on women’s demographics, height, weight, and method of delivery. Blood samples (4–5 mL) were collected for maternal thyroid function measurement. Maternal spot urine and breast milk, and spot infant urine samples were collected. Procedures were explained in detail to participating lactating women. Each mother-child pair was guided by the same professional staff member throughout the study process. Study staff was available to answer subjects’ questions and were responsible for the quality control of the sample collection.

### Breast milk sample collection

Subjects were asked to use a breast pump to empty their breasts before starting the study. The times of breast-feeding were not controlled. Breast milk samples were collected before and after each lactation. A breast milk sample collected after 8:00 am each day was the first sample of the day and samples were sequentially collected and recorded thereafter. Eight of 25 subjects recorded the exact time of each breastfeeding. Lactating women were required to retain no less than 2 mL of breast milk samples before and after each breastfeeding. The sample number and collection status were checked by study staff daily. Samples were stored at −20°C and tested within 2 weeks after collection.

### Dietary sample collection

The duplicate portion method was adopted to collect dietary specimens for seven consecutive days. Diet data were collected at the same time as the 24-h urine samples, all for 24 h. During the study, all the subjects were provided with a kitchen scale and required to accurately weigh all foods they ate. Duplicates of foods and beverages consumed during the day were systematically collected to determine and calculate daily dietary iodine intake. In order to further ensure the accuracy of dietary specimen retention, each subject was required to fill out a daily dietary record. Investigators collected and classified food samples and checked them against the dietary record forms each morning. Food samples were stored in vacuum sealed bags and transported to the laboratory. In the laboratory, food samples were weighed, mashed and homogenized. The homogenized 5–10 g samples were stored in a freezer at −20°C until analysis.

### Drinking water sample collection

The drinking water intake assessment included only pure water or tap water for drinking. Homemade hot beverages such as coffee, milk, tea, etc., are retained and measured separately for their iodine content and are classified as food iodine. Each participant was provided with a calibrated drinking glass. The amount of water consumed within 24 h was recorded. Five ml drinking water samples were retained, stored at 4°C and tested within 2 weeks. The daily iodine intake from drinking water was calculated.

### Urine sample collection

Every participant was provided with a 2.5-liter iodine-free plastic drum and a beaker with a handle. 24-h urine specimens were collected from 8:00 am to 8:00 am the next day. All urine was collected in plastic buckets. Investigators measured the volume of the urine samples. After mixing, 5 ml urine aliquots were stored at 4°C, and tested within 2 weeks. The 24-h urinary iodine excretion was obtained by multiplying the 24-h urinary iodine concentration by the 24-h urine excretion volume. Spot urine samples from mothers and infants were collected once using urine cups and urine collection bags one day before the start of the iodine metabolism study.

### Laboratory analyses

Breast milk iodine concentration (BMIC) was analyzed by inductively coupled plasma mass spectrometry (ICP-MS; iCAP Q, Thermo Fisher Scientific). ICP-MS was operated with argon (> 99.999%, high purity) in kinetic energy discrimination for the determination of elemental iodine. The flow rate of argon gas was set as follows: auxiliary: 0.8 L/min; nebulizer: 1.11 L/min; cooling gas flow rate: 14.00 L/min. The isotopes used for the measurements were 127I and 130Te. The standard reference material for whole milk powder (NIST 1549a; National Institute of Standards and Technology) was used for quality control. The intra- and inter- assay CVs were 1.27 and 2.95%, respectively. ICP-MS was also used for determination of water iodine concentration (WIC), food iodine, and urine iodine. Water samples were diluted 11-fold with 7 mmol/L hydrous ammonium and 100 μg/L Te. The inter-assay and intra-assay CV for drinking water measurement were 1.8–3.0 and 0.7–2.3%, respectively. Food samples were digested with 25% tetramethylammonium hydroxide at 90°C for 5 h. For food iodine samples, the standardized reference materials were purchased from National Institute of Standards and Technology (SRM 1548a for typical diet). The mean iodine concentration assessed by the method was 0.733 ± 0.035 mg/kg, compared to the reference value of 0.759 ± 0.103 mg/kg. The inter-assay and intra-assay CV were 1.5–2.6 and 2.8–4.0%, respectively. The analytical recovery from food was 98–110%. Urine samples were diluted 21-fold with 0.25% TMAH, 0.02% Triton X-100, and 100 μg/L Te before analysis using ICP-MS. The total inter-assay and intra-assay CV% for urinary iodine concentration (UIC) measurements were 1.4–3.2 and 0.6–1.8%, respectively. Thyroid function parameters, including serum free triiodothyronine (FT3), free thyroxine (FT4), and thyroid-stimulating hormone (TSH), were determined using the ADVIA Centaur CP Immunoassay System (Siemens). The lowest detection limits of TSH, FT4, and FT3 were 0.008 mIU/L, 1.3 and 0.3 pmol/L, respectively. Quality-control processes were performed according to the manufacturer’s instructions before, during, and after the testing. The intra-assay CVs for serum TSH, FT4 and FT3 were 2.1–4.9, 1.7–4.2, 2.4–3.1%, respectively. The inter-assay CVs for serum TSH, FT4 and FT3 were 1.5–4.4, 1.4–3.1, and 2.8–4.1%, respectively.

### Statistical analyses

Total iodine intake (TII) is the sum of water iodine and dietary iodine. We summarize normally distributed data as mean ± SD and non-normally distributed data as the median [quartile (Q)1–Q3 range]. Wilcoxon matched-pairs signed-ranks test was used to assess differences in BMIC before and after lactation. Spearman rank correlation was used to examine the correlations between BMIC and TII, UIC, and UIE. The estimated sample sizes with specified accuracy were calculated by the formula: N = (Z × CV/D)^2^ (10). The coefficient of variation (CV) was the standard deviation divided by the mean, as a proportion. The precision range (D) used to estimate the required sample size at precision levels varied from ± 1% to ± 50% (10, 11). The confidence interval (CI) value was set to 95%, the corresponding *Z* value was 1.96. A sensitivity analysis was performed to determine changing trends in BMIC by infant age. Physical development and BMIC consumed by infants of different weeks of age were compared using non-parametric Kruskal-Wallis tests. Linear mixed-effects models with per-subject random intercept were performed using PROC MIXED in SAS software for the longitudinal analyses of the factors associated with breast milk iodine. The following variables entered the final model separately as fixed effects: lactating women’s BMI, infants’ weeks of age, height and weight, TII, 24-h UIE, 24-h UIC, and the prior day’s TII, 24-h UIE, and 24-h UIC. We subsequently adjusted for the mother’s age, parity and the infant’s sex in the analysis of the baseline independent variables. For repeated independent variables, we adjusted for the following potential confounders: maternal age, parity, and BMI and the infants’ sex, gestational age at birth, birth height, birth weight, weeks of age, and current height and weight. Missing data were not included in the analysis. Analyses were performed using SAS statistical software version 9.4 (SAS Institute Inc., Cary, NC, USA).

## Results

### Baseline data

An iodine metabolism study was performed for seven consecutive days. One of the subjects underwent the experiment for 5 days. None of the subjects consumed sea fish, kelp or iodized salt during the experiment. Baseline characteristics are shown in Table 1. Lactating women had a mean age of 30.6 ± 6.3 years and all had normal thyroid function. The median (Q1, Q3) of TII, 24-h UIC, and 24-h UIE were 155 (90.0, 302) μg/d, 89.5 (55.7, 134) μg/L, and 106 (65.4, 162) μg/d, respectively. The infants, including 13 boys and 12 girls, aged 15.1 ± 4.9 weeks, were born *via* vaginal delivery and were exclusively breastfed. The median (Q1, Q3) of BMIC was 115 (86.7, 172) μg/L. The mothers’ median spot urinary iodine concentration was significantly lower than that of the infants. The infants’ length, weight and head circumference increased significantly with age (Supplementary Table 1). A gradual decline in median BMIC was observed from 5 to 26 weeks postpartum (*P* for trend = 0.04).

**TABLE 1:** Characteristics of lactating women and their infants.

Variables	
mother-infant pairs (n)	25
**lactating women**	
Age, years	30.6 ± 6.3
BMI, kg/m^2^	24.9 ± 3.0
Exclusive breastfeeding, n (%)	25 (100)
**Number of pregnancies**	
1, n (%)	6 (24.0)
2, n (%)	11 (44.0)
3, n (%)	7 (28.0)
4, n (%)	1 (4.0)
**Parity**	
1, n (%)	8 (32.0)
2, n (%)	14 (56.0)
3, n (%)	3 (12.0)
FT3, pmol/L	4.7 ± 0.4
FT4, pmol/L	13.2 ± 1.7
TSH, mIU/L	1.6 ± 0.8
WI, mL/d	1357 (900, 2053)
TII, μg/d	155 (90.0, 302)
Spot UIC, μg/L	93.0 (50.1, 169)
24-h UIC, μg/L	89.5 (55.7, 134)
24-h UIE, μg/d	106 (65.4, 162)
**Infants**	
**Gender**	
Boys, n (%)	13 (52.0)
Girls, n (%)	12 (48.0)
Weeks of age	15.1 ± 4.9
Birth length, cm	50.0 ± 0.9
Birth weight, kg	3.5 ± 0.5
Current length, cm	63.8 ± 4.0
Current weight, kg	7.3 ± 1.1
Spot UIC, μg/L	195 (138, 301)
Mean BMIC, μg/L	118 (90.1, 181)
Median BMIC, μg/L	115 (86.7, 172)

Data are presented as ratios or means ± SDs or median (Q1, Q3). BMI, body mass index; BMIC, breast milk iodine concentration; FT3, free triiodothyronine; FT4, free thyroxine; TSH, thyroid stimulating hormone; TII, total iodine intake; UIC, urinary iodine concentration; UIE, urinary iodine excretion; WI, water intake.

### Correlations and differences in breast milk iodine concentration before and after lactation

A total of 1,471 pairs of breast milk samples before and after breastfeeding were collected (Table 2). The BMIC concentrations before and after each feeding were closely correlated. A Wilcoxon matched-pairs signed-ranks test found that the BMIC before lactation [120 (85.3, 190) μg/L] was generally significantly higher than that after lactation [112 (81.6, 174) μg/L] (*P* < 0.001). However, the difference between before- and after-feeding BMIC was inconsistent in different feeding durations. The difference in BMIC between before-feeding and after-feeding was sometimes significant and sometimes not significant during a single feeding.

**TABLE 2:** Difference and correlation of breast milk iodine concentration (BMIC) before and after lactation.

Code	Pairs (n)	Before-BMIC (μg/L)	After-BMIC (μg/L)	*CC*	*p* ^#^
IB01	58	50.0 (43.6, 58.8)	50.6 (44.1, 59.2)	0.95*	0.17
IB02	84	128 (103, 153)	126 (109, 152)	0.74*	0.32
IB03	40	95.6 (79.0, 114)	93.9 (77.7, 113)	0.99*	0.002
IB04	62	111 (92.5, 152)	97.9 (81.7, 143)	0.89*	<0.001
IB05	76	126 (107, 145)	109 (94.8,134)	0.93*	<0.001
IB06	82	94.7 (80.7, 112)	94.0 (79.6, 108)	0.93*	0.37
IB07	73	56.7 (45.7, 69.8)	54.4 (44.9, 69.1)	0.90*	0.005
IB08	67	105 (92.4, 124)	97.1 (86.4, 111)	0.87*	<0.001
IB09	35	591 (397, 698)	496 (393, 603)	0.89*	0.03
IB10	51	179 (140, 243)	162 (123, 211)	0.88*	<0.001
IB11	101	297 (212, 365)	327 (176, 405)	0.80*	0.10
IB12	59	77.4 (56.1, 118)	74.8 (54.7, 112)	0.95*	0.001
IB13	28	184 (160, 216)	161 (148, 203)	0.87*	<0.001
IB14	48	116 (110, 133)	109 (102, 117)	0.42*	0.001
IB15	61	74.1 (62.8, 84.1)	71.1 (58.0, 82.3)	0.88*	0.003
IB16	37	156 (112, 177)	139 (102, 186)	0.86*	0.06
IB17	61	69.6 (61.6, 83.4)	70.5 (59.1, 82.0)	0.86*	0.96
IB18	35	94.6 (84.9, 107)	94.3 (74.0, 106)	0.83*	0.29
IB19	65	121 (101, 219)	154 (102, 238)	0.51*	0.10
IB20	78	701 (572, 797)	743 (608, 832)	0.70*	<0.001
IB21	56	126 (111, 134)	125 (107, 140)	0.66*	0.74
IB22	58	153 (128, 190)	118 (106, 144)	0.83*	<0.001
IB23	66	94.9 (60.1, 149)	90.5 (51.7, 135)	0.90*	0.002
IB24	64	214 (185, 243)	182 (152, 209)	0.69*	<0.001
IB25	26	127 (117, 169)	128 (111, 155)	0.20	0.42
Total	1471	120 (85.3, 190)	112 (81.6, 174)	0.94*	<0.001

Data are presented as medians (25th, 75th percentiles). After-BMIC, breast milk iodine concentration after a feeding; Before-BMIC, breast milk iodine concentration before a feeding; CC, correlation coefficient. CC was calculated by the Spearman Rank correlation test.

^#^Made by the Wilcoxon matched-pairs signed-ranks test.

*Indicates statistical significance (*P* < 0.05).

### Correlations between breast milk iodine concentration and maternal iodine intake and urine iodine

Table 3 shows the correlations between BMIC at each time point and total maternal iodine intake, UIE and UIC. BMIC was positively correlated with TII, UIE, and UIC on the same day or in the previous day. The BMIC gradually became more correlated with the TII and urine iodine over time on the same day. However, the correlation with the previous day’s iodine intake and urine iodine gradually became smaller as each day progressed. The correlation between BMIC and 24-h UIE was greatest, suggesting that the excretion of breast milk iodine is similar to that of urine iodine. In addition, the correlation between BMIC and TII the prior day was higher than that on the same day when for the first three feedings of each day, which indicates that BMIC in the morning is strongly influenced by the prior day’s TII.

**TABLE 3:** Correlations between breast milk iodine concentration (BMIC) and total iodine intake (TII), urinary iodine excretion (UIE) and urinary iodine concentration (UIC) on the same day and the prior day.

BMIC collection times	Same day							Prior day						
	*N*	TII		24-h UIC		24-h UIE		*N*	TII		24-h UIC		24-h UIE	
		CC	*P*	CC	*P*	CC	*P*		CC	*p*	CC	*P*	CC	*P*
1	170	0.50	<0.001	0.55	<0.001	0.65	<0.001	146	0.62	<0.001	0.63	<0.001	0.72	<0.001
2	168	0.49	<0.001	0.55	<0.001	0.66	<0.001	143	0.57	<0.001	0.62	<0.001	0.70	<0.001
3	171	0.56	<0.001	0.58	<0.001	0.70	<0.001	146	0.59	<0.001	0.61	<0.001	0.71	<0.001
4	170	0.60	<0.001	0.63	<0.001	0.70	<0.001	145	0.55	<0.001	0.60	<0.001	0.67	<0.001
5	170	0.64	<0.001	0.63	<0.001	0.72	<0.001	145	0.54	<0.001	0.53	<0.001	0.62	<0.001
6	156	0.61	<0.001	0.67	<0.001	0.72	<0.001	133	0.55	<0.001	0.47	<0.001	0.53	<0.001
7	144	0.56	<0.001	0.63	<0.001	0.73	<0.001	123	0.55	<0.001	0.44	<0.001	0.54	<0.001
8	129	0.51	<0.001	0.62	<0.001	0.72	<0.001	110	0.50	<0.001	0.44	<0.001	0.52	<0.001
9	100	0.52	<0.001	0.56	<0.001	0.75	<0.001	83	0.49	<0.001	0.34	0.002	0.50	<0.001
10	68	0.71	<0.001	0.59	<0.001	0.84	<0.001	57	0.56	<0.001	0.25	0.06	0.57	<0.001
11	49	0.73	<0.001	0.48	0.001	0.85	<0.001	42	0.56	0.0001	0.30	0.05	0.64	<0.001
N ≥ 12	69	0.75	<0.001	0.56	<0.001	0.91	<0.001	55	0.75	<0.001	0.33	0.01	0.69	<0.001
Total^a^	1564	0.58	<0.001	0.59	<0.001	0.72	<0.001	1328	0.57	<0.001	0.51	<0.001	0.63	<0.001
Total^b^	173	0.60	<0.001	0.65	<0.001	0.76	<0.001	148	0.57	<0.001	0.55	<0.001	0.64	<0.001
Total^c^	173	0.62	<0.001	0.64	<0.001	0.74	<0.001	148	0.57	<0.001	0.55	<0.001	0.64	<0.001

^a^Based on all breast milk samples. BMIC is the average breast iodine concentration before and after lactation. ^b^BMIC is the mean iodine concentration of the breast milk samples for a given a day. ^c^BMIC is the median iodine concentration of the breast milk samples for a given a day. BMIC, breast milk iodine concentration; CC, Correlation Coefficient; TII, total iodine intake; 24-h UIC, 24-h urinary iodine concentration; 24-h UIE, 24-h urinary iodine excretion.

### Optimal sampling time of breast milk iodine

The median BMIC is often used to represent a day’s milk iodine level. Table 4 presents the correlation coefficients between the BMIC at each time point and the median or mean BMIC in a day. Estimating the optimal collection time based on breastfeeding times is only a rough estimate. We can see that the median iodine concentration of milk samples collected at the fourth and fifth feeds and after the 12th feed of the day are closest to the median BMIC (*r* = 0.96). Similarly, we found that the BMIC for samples collected at the fourth and fifth feedings, and after the 10th feeding was closest to the median BMIC of the day in the eight lactating mothers who accurately recorded the sampling time. These two time points are roughly in the afternoon or after midnight.

**TABLE 4:** Estimation of the optimal sampling time of breast milk iodine.

BMIC collection times	Twenty-five subjects^a^					BMIC collection times	Eight subjects^b^				
		Mean BMIC		Median BMIC				Mean BMIC		Median BMIC	
	*N*	in a day		in a day			*N*	in a day		in a day	
		CC	*P*	CC	*P*			CC	*P*	CC	*P*
1	170	0.87	<0.001	0.88	<0.001	1	56	0.87	<0.001	0.88	<0.001
2	168	0.88	<0.001	0.88	<0.001	2	55	0.88	<0.001	0.87	<0.001
3	171	0.92	<0.001	0.94	<0.001	3	56	0.94	<0.001	0.94	<0.001
4	170	0.94	<0.001	0.96	<0.001	4	56	0.94	<0.001	0.96	<0.001
5	170	0.94	<0.001	0.96	<0.001	5	54	0.92	<0.001	0.95	<0.001
6	156	0.95	<0.001	0.93	<0.001	6	54	0.91	<0.001	0.89	<0.001
7	144	0.94	<0.001	0.89	<0.001	7	52	0.92	<0.001	0.88	<0.001
8	129	0.93	<0.001	0.88	<0.001	8	49	0.87	<0.001	0.82	<0.001
9	100	0.94	<0.001	0.91	<0.001	9	39	0.92	<0.001	0.89	<0.001
10	68	0.95	<0.001	0.95	<0.001	N ≥ 10	47	0.94	<0.001	0.95	<0.001
11	49	0.91	<0.001	0.92	<0.001						
N ≥ 12	69	0.96	<0.001	0.96	<0.001						

^a^Twenty-five subjects recorded the number of feedings and collected breast milk before and after each feeding. ^b^Eight of twenty-five subjects recorded the time of each breastfeeding and collected breast milk before and after each feeding. The BMIC for each lactation is the average BMIC before and after lactation. The mean BMIC in a day is the mean iodine concentration of the breast milk samples for a given day. The median BMIC in a day is the median iodine concentration of the breast milk samples for a given day.

### Variation in breast milk iodine

We evaluated the BMIC variation from multiple perspectives (Table 5). The mean, median, and lowest and highest CV for BMIC in an individual were 34.3, 31.4, 22.2, and 57.1%, respectively. These values were 19.1, 15.5, 1.15, and 78.6%, respectively, in an individual on a single day. The population median (Q1, Q3) of CV in BMIC was 93.2% (91.1, 102%). The median variation in BMIC consumed by infants was lower than maternal 24-h UIC, 24-h UIE, and TII. However, the population median variation of 24-h UIC and BMIC is similar.

**TABLE 5:** Variation in iodine nutrition indicators.

CV	BMIC (μg/L)			24-h UIC (μg/L)		24-h UIE (μg/d)		TII (μg/d)	
	A population	An individual	An individual a day	A population	An individual	A population	An individual	A population	An individual
Min	78.0%	22.2%	1.15%	60.7%	11.0%	54.2%	9.2%	106%	12.5%
Mean	99.4%	34.3%	19.1%	90.9%	38.5%	74.0%	35.3%	118%	54.6%
Q1	91.1%	27.3%	11.4%	78.0%	27.8%	66.3%	28.9%	108%	28.5%
Median	93.2%	31.4%	15.5%	94.2%	41.3%	77.1%	33.1%	114%	52.1%
Q3	102%	37.2%	22.9%	99.9%	46.8%	80.3%	43.9%	128%	76.2%
Max	139%	57.1%	78.6%	125%	73.1%	93.4%	60.6%	133%	107%

BMIC, breast milk iodine concentration; CV, coefficient of variation; 24-h UIC, 24-h urinary iodine concentration; 24-h UIE, 24-h urinary iodine excretion.

**FIGURE 2:**
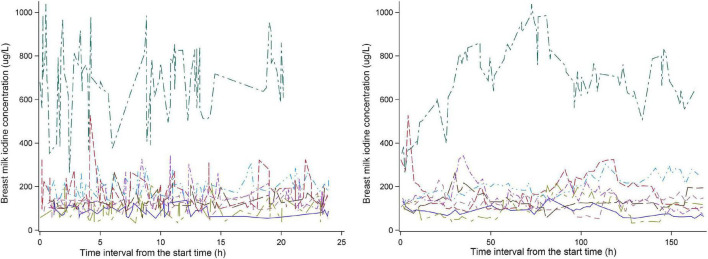
The changing trend of breast milk iodine concentration in eight lactating women.

Eight of twenty-six subjects recorded the time of each breastfeeding. The change in BMIC after 8:00 am on the first day of the experiment is shown in Figure 2. We can see that the BMIC levels in most lactating mothers fluctuate relatively smoothly. One lactating mother had a particularly high level of BMIC. In addition, we estimated each breastfeeding time for the remaining 18 lactating mothers based on the number of breastfeeds per day. The fluctuations in BMIC in all subjects are shown in Supplementary Figure 1.

### Sample sizes for iodine nutrition monitoring

Table 6 lists the number of samples necessary to estimate breast milk iodine status in a population, an individual, and an individual day with 95% confidence. The sample size varies with the different CVs and precision ranges. Specifically, in a study of 334 participants providing one breast milk sample, the precision range will be about ± 10%. This means that, if the mean BMIC in a survey was 150 μg/L, the true mean BMIC in that population would be between 135 and 165 μg/L. According to the median CV, obtaining a precision range of ± 10% in an individual would require thirty-eight breast milk samples. Furthermore, if we want to estimate the iodine content of a person’s breast milk on a given day, collecting two samples from an individual in a day would achieve a ± 20% precision range.

**TABLE 6:** Number of samples needed to be 95% confident of being within a specified range for estimating the breast milk iodine status.

Precision ranges	A population			An individual			An individual a day		
	Median CV	Lowest CV	Highest CV	Median CV	Lowest CV	Highest CV	Median CV	Lowest CV	Highest CV
± 1%	33369	23372	74224	3788	1893	12525	923	5	23733
±2%	8342	5843	18556	947	473	3131	231	1	5933
± 5%	1335	935	2969	152	76	501	37	1	949
±10%	334	234	742	38	19	125	9	1	237
± 20%	83	58	186	9	5	31	2	1	59
±30%	37	26	82	4	2	14	1	1	26
± 40%	21	15	46	2	1	8	1	1	15
±50%	13	9	30	2	1	5	1	1	9

Calculated from N = (Z × CV/D)^2^, where *Z* = 1.96 for 95% CI and D is the precision range. CV, coefficient of variation.

### Parameters associated with breast milk iodine

A mixed-effects regression analysis was used to analyze the factors associated with BMIC (Table 7). Infant week of age, height, and weight were not related to BMIC. Maternal TII and urine iodine were significantly correlated with BMIC. In particular, the maternal 24-h UIE was found to be the best predictor for the level of BMIC (β = 0.71, 95% CI: 0.64, 0.79). In addition, the prior day’s TII (β = 0.06, 95% CI: 0.03, 0.09), 24-h UIC (β = 0.20, 95% CI: 0.10, 0.30) and 24-h UIE (β = 0.32, 95% CI: 0.23, 0.41) were related to the day’s BMIC.

**TABLE 7:** Linear mixed-effects models of predictors of breast milk iodine concentration (BMIC).

Variables	Unadjusted			Adjusted		
	β	95% CI	*p*	β	95% CI	*p*
Lactating women’s BMI^a^	15.5	−3.92, 35.0	0.12	17.2	−4.07, 38.5	0.11
Infant’s weeks of age^a^	−3.79	−16.4, 8.80	0.56	−3.09	−17.2, 11.0	0.67
Infant’s height^a^	−5.64	−20.8, 9.48	0.47	−5.43	−22.8, 12.0	0.54
Infant’s weight^a^	−2.92	−36.6, 30.7	0.86	−1.37	−40.7, 38.0	0.95
TII^b^	0.09	0.06, 0.12	<0.001	0.08	0.05, 0.11	<0.001
24-h UIC^b^	0.54	0.46, 0.62	<0.001	0.54	0.46, 0.62	<0.001
24-h UIE^b^	0.72	0.65, 0.80	<0.001	0.71	0.64, 0.79	<0.001
the prior day’s TII^b^	0.07	0.03, 0.10	<0.001	0.06	0.03, 0.09	<0.001
the prior day’s 24-h UIC^b^	0.20	0.11, 0.30	<0.001	0.20	0.10, 0.30	<0.001
the prior day’s 24-h UIE^b^	0.33	0.25, 0.42	<0.001	0.32	0.23, 0.41	<0.001

^a^Adjusted for the mother’s age, parity, and the infant’s sex.

^b^Adjusted for the mother’s age, parity, BMI and the infant’s sex, gestational age at birth, birth height, birth weight, weeks of age, current height and weight.

## Discussion

Compared with other age groups, neonates and infants are susceptible to iodine deficiency as they have lower intrathyroidal iodine storage and the highest iodine requirements relative to body weight (9–12). Breast milk is the only iodine source for exclusively breastfed infants. Appropriate breast milk iodine levels are required for infants’ normal physical and neurologic growth and maturation (13). The mother’s body has physiological mechanisms for iodine regulation during lactation. Iodide transport and uptake by the mammary gland are mediated by the sodium-iodide symporter (NIS), the expression of which is increased in the lactating breast (14, 15). However, the breast’s ability to regulate iodine concentrations seems to be limited (16), as breast milk iodine concentrations vary widely (9). BMIC ranges of 5.4–2,529 μg/L have been reported, even in populations considered iodine sufficient (5, 7, 17–20).

Our study site has been regarded as iodine sufficient based on local drinking water iodine and urine iodine monitoring data. The median BMIC was 115 μg/L in our study. In general, the volume of breast milk ingested by infants aged 0–6 months is about 500–1,000 ml/day, similar to what we observed. The Chinese iodine Adequate Intake (AI) for infants aged 0–6 months was based upon a median BMIC of 112 μg/L multiplied by an average milk excretion of 0.75 L/day to approximately equal 85 μg/day (21). The infant iodine AI defined by the U.S. Institute of Medicine (IOM) was 110 μg/day and the recommended infant iodine intake defined by the WHO was 90 μg/day. If the optimal BMIC is inferred from the recommended iodine intake and a breast milk volume of 500–1,000 ml/day, the normal BMIC range is 85–220 μg/L.

A trend toward a moderate decrease in BMIC over the course of lactation has been found in longitudinal studies in iodine-deficient areas (22, 23), iodine-sufficient areas (8, 24), and iodine-excessive areas (25). We also observed a gradual decrease in median BMIC with increasing infant age and body mass from 5 to 26 weeks postpartum. In addition, we found that BMIC before each feeding was generally higher than that after (*P* < 0.001), indicating there is a dynamic change in BMIC during each breastfeeding. When Dold et al. required subjects to provide three consecutive breast milk samples from one feeding session, they similarly found that iodine content was significantly lower in the post-feeding samples compared to the pre-and mid-feeding breast milk samples (26). A previous study pointed out that the lower BMIC in the post-feeding period may be the result of physiological changes in the composition of breast milk during feeding. There is a decrease in the proportion of the iodine-containing aqueous phase due to an increase in the fat content of breast milk at the end of feeding (27). Although some studies have found no differences in BMIC before and after lactation (7, 28, 29), paired tests in our study demonstrated that BMIC sometimes does differ significantly before and after lactation. We speculate that the differences in milk iodine concentration before and after a feeding may be related to the duration of a single feeding.

Moreover, we described diurnal changes in BMIC. The correlations between BMIC collected after 8:00 am and the day’s TII, 24-h UIE, and 24-h UIC gradually increased over the course of each day. In contrast, the correlation with the prior day’s TII and urinary iodine level progressively decreased. BMIC was more highly correlated with 24-h UIE than with TII and 24-h UIC, which is consistent with the results of a prior Danish study (30). Although the changing patterns of BMIC are similar to those of urinary iodine, BMIC level is primarily determined by the maternal dietary iodine intake (16, 31, 32). BMIC responds quickly to recent changes of dietary iodine intake (within hours), either from supplements or food (30, 33). The ingested iodine is rapidly excreted in breast milk, and the BMIC peaks within 6 h after consumption (16, 33).

Substantial BMIC day-to-day intra-individual variability (1.15–78.6%) was observed in our study. Kirk et al. described considerable diurnal variation in BMIC, but no specific variability values were given (34). A recent review found no consistent trends in BMIC by the time of day (9), although our study demonstrates irregular diurnal fluctuations in BMIC. Overall, we found the population variability of BMIC was significantly higher than the inter-individual variability. According to prior studies, BMIC is widely variable across populations, which is likely influenced by habitual and recent maternal iodine intake and status (9). In our study, the variability in TII was the largest, which is consistent with the findings of Chen et al. (35). The variability of BMIC and urinary iodine within the population and for each individual were roughly similar, which confirms that the excretion rhythms of milk and urinary iodine are similar. The number of breast milk samples needed to estimate the iodine level in a population in a day with 95% confidence within a precision range of ± 20% was about two. We recommend collecting samples after lunch or after midnight. To estimate an individual’s iodine level with 95% confidence within a precision range of ± 20%, the breast milk sample size required (9 samples) is lower than that of spot urine (14 samples) (36). Although urinary iodine concentration is commonly used to assess population iodine nutritional status, BMIC may be the preferred index for infants (6, 37).

In this study, maternal 24-h UIE was found to be the best predictor for the level of BMIC. The positive correlation between BMIC and 24-h UIE was stronger than the correlation with dietary iodine intake. Accordingly, we speculate that the iodine excretion function of the mammary gland is similar to that of the kidney under specific conditions. This excretion mechanism is relatively stable in a well-nourished population. Several studies have found the positive correlation between BMIC and creatinine-adjusted UIC is stronger in iodine-sufficient populations (9, 30). BMIC has been shown to be independent of maternal fluid intake (30, 38, 39), while the UIC is affected by it.

### Strengths and limitations

This is the first study to comprehensively summarize characteristics and predictors of BMIC based on a repeated measures study of iodine metabolism. We accurately measured dietary iodine intake and 24-h urinary iodine excretion. Each nursing mother collected all milk samples before and after each feeding for each day, and eight of them recorded the time of each feeding. The differences in BMIC before and after each feeding as well as the diurnal variation trends and individual and population variability of BMIC were determined. Additionally, the representative optimal sampling time was proposed, and the significant related factors of breast milk iodine level were identified.

Our study has some limitations. First, sampling time was not recorded by all participants. Estimates of sampling time in this study were based on samples collected from 8 of the 25 subjects. Although we roughly estimated the sampling time according to the daily number of samples, the optimal sampling time for BMIC needs to be verified by future studies. Second, we did not record the time between the previous meal of the mother and the milk sampling. Third, the sample size of this study was relatively small. In estimating of the appropriate ranges for BMIC, we only considered that the infant was healthy and the iodine intake was appropriate, and we did not explore reasons for high or low milk iodine concentrations. Large-scale population iodine nutrition epidemiological surveys, including in populations with thyroid diseases, will be needed to clarify the reasons for the large variations in BMIC.

## Conclusion

The correlation between BMIC and daily iodine intake and urinary iodine increased gradually over the course of each day. Breast milk samples collected in the afternoon or after midnight are closest to the median breast milk iodine level throughout the day. BMIC was significantly associated with recent iodine intake, with an excretion pattern similar to 24-h UIE.

## Data availability statement

The original contributions presented in this study are included in the article/Supplementary material, further inquiries can be directed to the corresponding author.

## Ethics statement

The studies involving human participants were reviewed and approved by Ethics Committee of Tianjin Medical University. Written informed consent to participate in this study was provided by the participants’ legal guardian/next of kin.

## Author contributions

WG: visualization, investigation, writing—original draft, funding acquisition, and formal analysis. WW and SL: investigation and writing—original draft. EP and ZR: writing—original draft. MG: investigation and data curation. ZP, NZ, and KZ: investigation. YY: conceptualization, supervision, and investigation. WZ: funding acquisition and project administration. All authors contributed to the article and approved the submitted version.
