# Supplementary material for: Characteristics and predictors of breast milk iodine in exclusively breastfed infants: Results from a repeated-measures study of iodine metabolism
Source: Front Nutr. 2022 Nov 9;9:1017744. doi: 10.3389/fnut.2022.1017744 (PMC9682142; doi:10.3389/fnut.2022.1017744)
Supplement: Supplementary file 1 [file Table_1.DOCX]

Supplementary Material

# Supplementary Table

**Supplementary Table 1.** Physical development and breast milk iodine levels in infants at different weeks of age

| Variables | weeks of age | | | *P* | *P trend* |
| --- | --- | --- | --- | --- | --- |
|  | 5-12 weeks | 13-18 weeks | 19-26 weeks |  |  |
| N | 7 | 12 | 6 |  |  |
| Current length, cm | 59.9 ± 3.2 | 64.4 ± 3.2 | 67.1 ± 3.0 | 0.002 | <0.001 |
| Current weight, kg | 6.2 ± 0.9 | 7.4 ± 0.6 | 8.3 ± 1.3 | 0.002 | <0.001 |
| Current headcir, cm | 39.6 ± 1.3 | 41.3 ± 2.3 | 43.1 ± 1.4 | 0.02 | 0.006 |
| Current chestcir, cm | 43.4 ± 5.5 | 46.3 ± 3.1 | 45.7 ± 2.2 | 0.28 | 0.31 |
| Mean BMIC, μg/kg | 141 (95.8, 220) | 111 (72.6, 149) | 107 (94.2, 148) | 0.43 | 0.07 |
| Median BMIC, μg/kg | 134 (87.3, 195) | 111 (68.3, 156) | 106 (94.6, 145) | 0.41 | 0.05 |
| Mean BMIC, μg/L | 148 (102, 230) | 115 (77.0, 153) | 113 (98.8, 152) | 0.42 | 0.06 |
| Median BMIC, μg/L | 136 (92.0, 208) | 115 (71.5, 161) | 111 (97.6, 142) | 0.41 | 0.04 |

BMIC, breast milk iodine concentration; chestcir, chest circumference; headcir, head circumference.

# Supplementary Figure


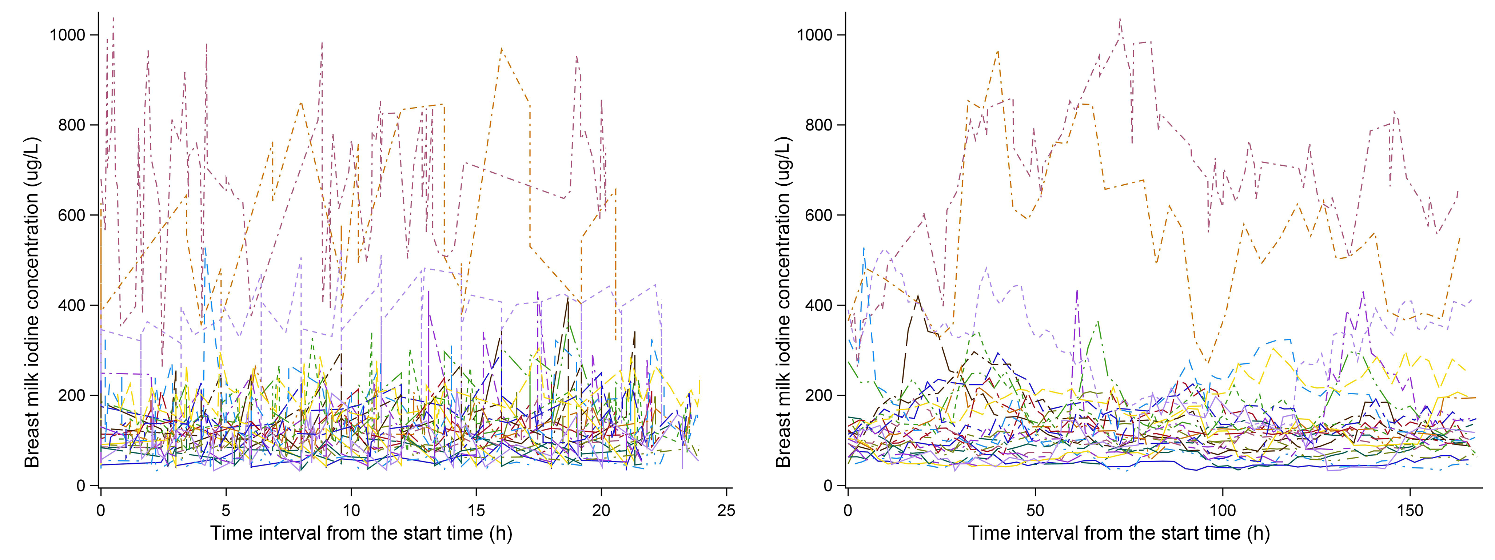


**Supplementary Figure 1.** The fluctuations of breast milk iodine concentration in all subjects
